# Early pastoral economies along the Ancient Silk Road: Biomolecular evidence from the Alay Valley, Kyrgyzstan

**DOI:** 10.1371/journal.pone.0205646

**Published:** 2018-10-31

**Authors:** William Taylor, Svetlana Shnaider, Aida Abdykanova, Antoine Fages, Frido Welker, Franziska Irmer, Andaine Seguin-Orlando, Naveed Khan, Katerina Douka, Ksenia Kolobova, Ludovic Orlando, Andrei Krivoshapkin, Nicole Boivin

**Affiliations:** 1 Department of Archaeology, Max Planck Institute for the Science of Human History, Jena, Germany; 2 Institute of Archaeology and Ethnography, Siberian Branch, Russian Academy of Sciences, Novosibirsk, Russia; 3 Altai State University, Barnaul, Russia; 4 American University of Central Asia, Bishkek, Kyrgyz Republic; 5 Laboratoire d’Anthropobiologie Moléculaire et Imagerie de Synthèse, Université de Toulouse, Université Paul Sabatier, Toulouse, France; 6 Centre for GeoGenetics, Natural History Museum of Denmark, Copenhagen, Denmark; 7 Natural History Museum of Denmark, University of Copenhagen, Copenhagen, Denmark; 8 Max Planck Institute for Evolutionary Anthropology, Leipzig, Germany; 9 Department of Biotechnology, Abdul Wali Khan University, Mardan, Pakistan; 10 Novosibirsk State University, Novosibirsk, Russia; Institucio Catalana de Recerca i Estudis Avancats, SPAIN

## Abstract

The Silk Road was an important trade route that channeled trade goods, people, plants, animals, and ideas across the continental interior of Eurasia, fueling biotic exchange and key social developments across the Old World. Nestled between the Pamir and Alay ranges at a baseline elevation of nearly 3000m, Kyrgyzstan’s high Alay Valley forms a wide geographic corridor that comprised one of the primary channels of the ancient Silk Road. Recent archaeological survey reveals a millennia-long history of pastoral occupation of Alay from the early Bronze Age through the Medieval period, and a stratified Holocene sequence at the site of Chegirtke Cave. Faunal remains were recovered from test excavations as well as surface collection of material from recent marmot activity. Although recovered specimens were highly fragmented and mostly unidentifiable using traditional zooarchaeological methods, species identification via collagen mass fingerprinting (ZooMS) coupled with sex and first-generation hybrid identification through ancient DNA enabled preliminary characterization of the animal economy of Alay herders. Our new results indicate primary reliance on sheep at Chegirtke Cave (ca. 2200 BCE), with cattle and goat also present. The discovery of a large grinding stone at a spatially associated Bronze or Iron Age habitation structure suggests a mixed agropastoral economic strategy, rather than a unique reliance on domestic animals. Radiocarbon-dated faunal assemblages from habitation structures at nearby localities in the Alay Valley demonstrate the presence of domestic horse, as well as Bactrian camel during later periods. The current study reveals that agropastoral occupation of the high-mountain Alay corridor started millennia before the formal establishment of the Silk Road, and posits that ZooMS, when paired with radiocarbon dates and ancient DNA, is a powerful and cost-effective tool for investigating shifts in the use of animal domesticates in early pastoral economies.

## Introduction

In the first millennium BC, the Trans-Eurasian system of caravan routes known as the Silk Road became one of the world’s most important channels of early globalization and transcontinental exchange, linking China with Central Asia and Europe. Although historical documents attest to the formalization of trade networks at the end of the first millennium BCE [1], archaeological research demonstrates that domestic plants from both the western and eastern reaches of Eurasia spread across the mountainous interior among pastoral societies during the Bronze and Early Iron Ages [2,3]. By the time of the western Han Dynasty, at the end of the first millennium BCE, domestic animals–particularly horses—were exchanged in the tens of thousands from highland areas of Central Asia to the Central Plains of China [3,4]. The “blood-sweating” or “heavenly” horses from the Ferghana Valley (comprising parts of modern-day Kyrgyzstan, Uzbekistan, and Tajikistan), mentioned in Sima Qian’s *Shiji*, are supposed to have descended from dragons, and were especially prized for their large size, angular build, and stamina [5]. In later centuries, annual trade in horses between China and Central Asia, in exchange for goods such as tea and silk, would reach into the hundreds of thousands [6,7]. Because of the prominent role interior Central Asia and its pastoral peoples played in the exchange of plant and animal domesticates, understanding the region’s early economy is particular important for the study of late Eurasian prehistory.

### Regional background

**Fig 1:**
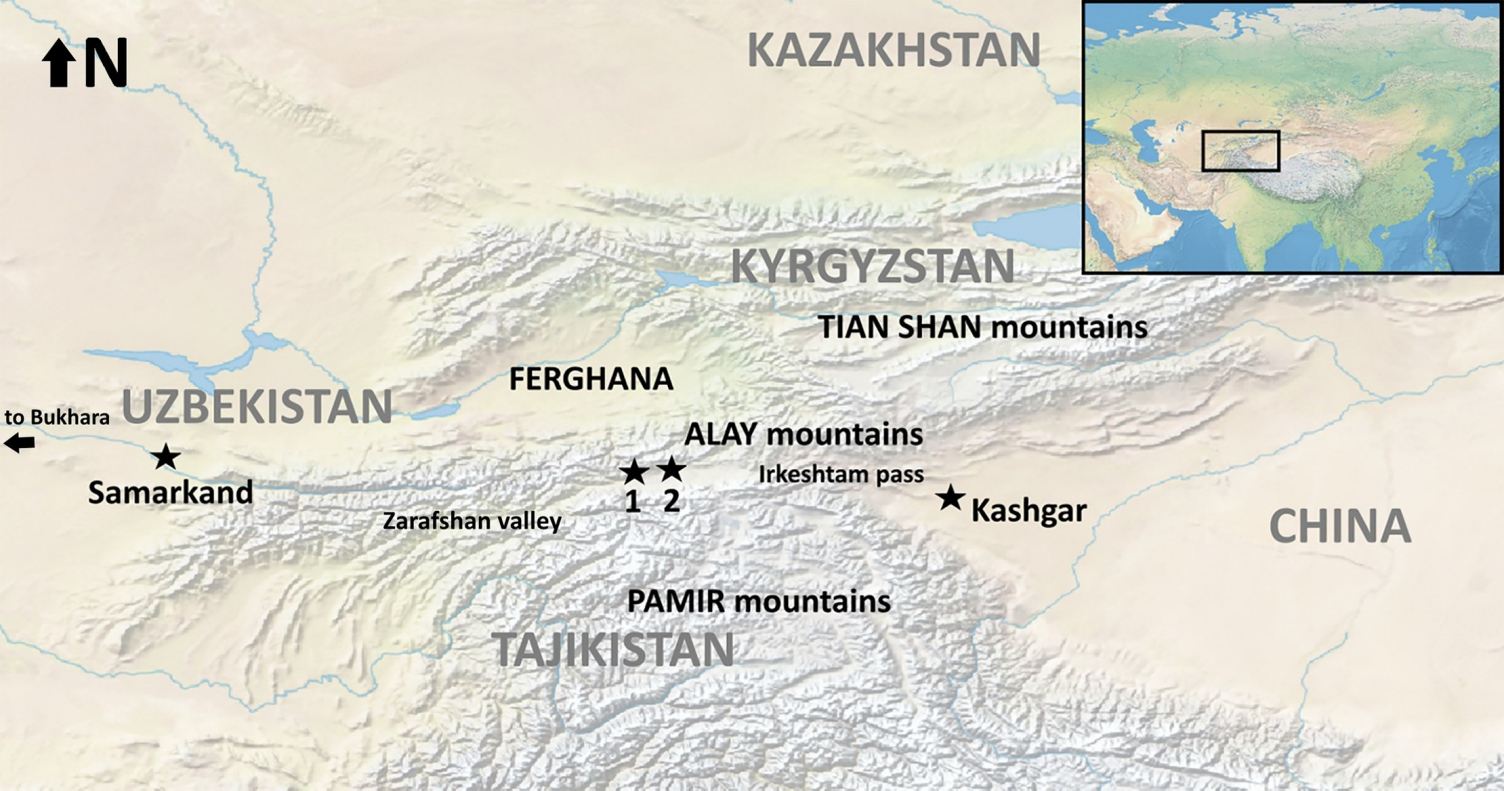
Map showing the study region (Alay Valley), as it relates to key geographic features. The sites addressed in the present study (1, Chegirtke Cave/Chegirtke Canyon/Kyzyl-Unkur, and 2, the Alay Site) are situated in the Alay Valley, nestled between the Alay and the Pamir mountain ranges in southern Kyrgyzstan.

Seated between the high Pamir Mountains and the Alay Range along the Tajik border (Fig 1), Kyrgyzstan’s Alay Valley is a key natural travel corridor. The landform, nearly 40 km wide in many places, links the rich Ferghana Valley, and the Zarafshan valley leading towards the oasis cities of Samarkand and Bukhara at its western terminus, with the city of Kashgar and the territory of modern China at its eastern limit, thereby forming an important channel of the ancient Silk Road[8]. Today, the region is home to a number of villages practicing a kind of collective herding system, in which many families’ animals are herded collectively and may follow either vertical or horizontal seasonal pasture movements [9], while some areas are cultivated agriculturally. Traditionally, livestock in Alay would be grazed on the warmer south-facing slopes during the winter months, while wetter north-facing slopes and the higher-altitude eastern reaches of the valley were preferred in the spring and summer [10,11]. Although sheep and goat dominate Kyrgyzstan’s livestock economy today, the Alay region is also home to lower numbers of cattle, yak, and camel. Due to the region’s rugged terrain, horses, mules, and donkey remain an important form of transport [10]. Until the early 1990’s, seasonal migrations of livestock linked Alay with the Ferghana Valley across the Shiman and Jangy Jer passes [11].

### Archaeological background

Although the ancient economy of the region that falls today within the state of Kyrgyzstan is poorly characterized [12], the Ferghana Valley has been inhabited since the Paleolithic period [13,14]. In Alay itself, the eponymous Alay site provides evidence that the valley was at least occasionally used by Paleolithic hunter-gatherers [15]. During the late 3^rd^ and early 2^nd^ millennium BCE, much of the region of Central Asia bracketing the Alay valley witnessed key changes in material culture, including the appearance of distinctive architecture, localized metal working, and “steppe” style ceramics [16]. Kohl [16] attributes this change to the migration of semi-sedentary, cattle-herding pastoralists spurred on by the emergence of horse or camel transport. In any case, archaeobotanical study of the site of Aygyrzhal-2 in central Kyrgyzstan shows that people practiced a kind of mixed agropastoralism–as evidenced by grains of wheat and barley, along with bones of domestic horse and sheep/goat interred in a ritual pit–by ca. 1600 BCE [17]. By the end of the first millennium BCE, the Alay valley served as a key travel corridor for merchant routes linking Ferghana with Kashgar and modern China through the Irshketam pass [8]. In recent years, scholars have recognized that patterns of landscape use by earlier pastoral groups likely influenced the development of these later trade networks [18]. Consequently, although analogy with the archaeological record of adjoining regions provides some hints at the nature of Bronze and Iron Age subsistence, understanding the formation of the Silk Road and the biological exchange along it requires a clearer view of the prehistoric economy of high-mountain trade corridors like the Alay Valley.

## Materials and methods

### Survey

**Fig 2:**
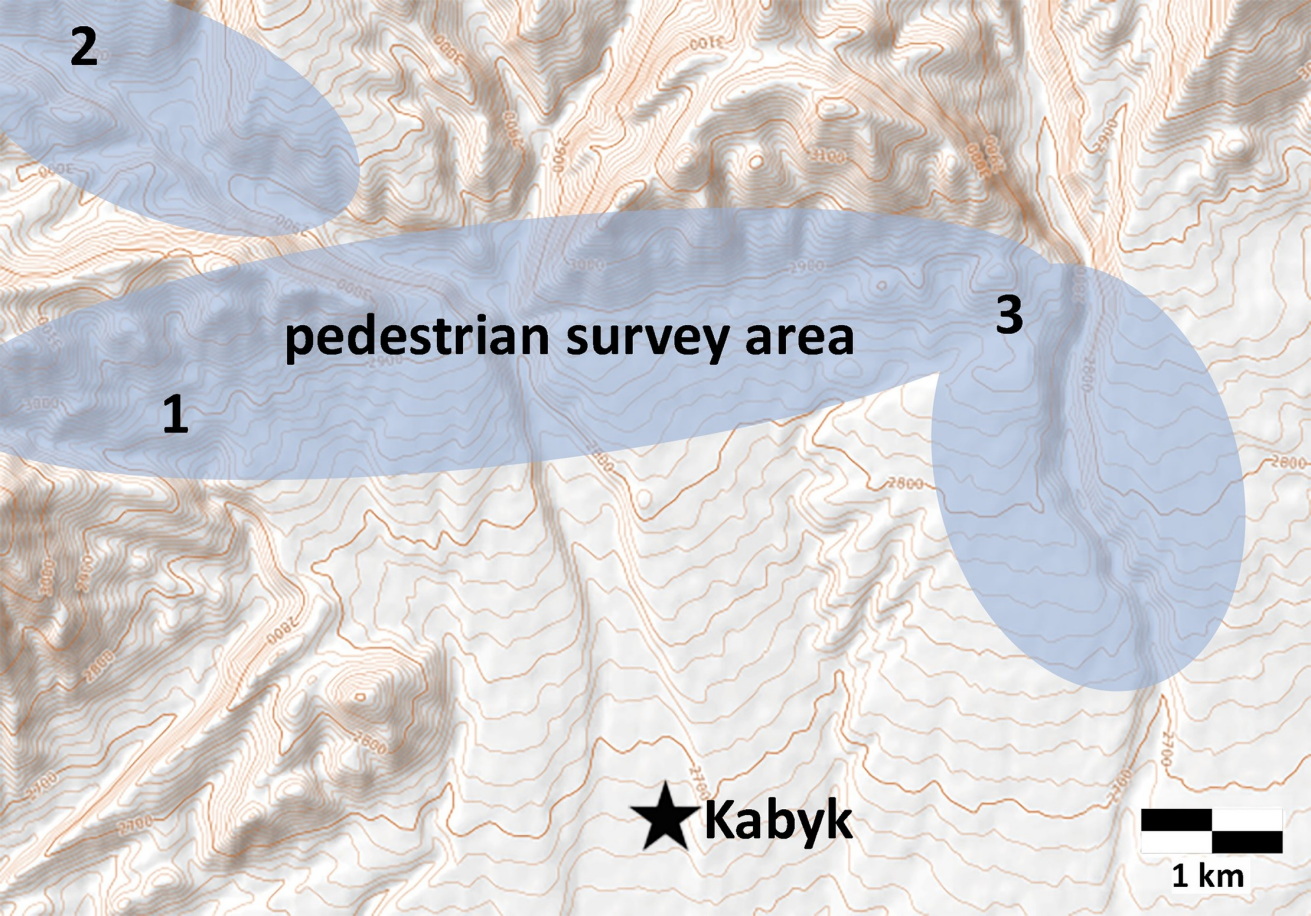
Pedestrian survey area north of Kabyk village, including locations of study sites: 1) Chegirtke Cave, 2) Chegirtke Canyon, and 3) Kyzyl-Unkur.

In 2017, a joint international expedition between the Russian Academy of Sciences-Siberian Branch and the American University of Central Asia carried out archaeological survey in the western part of the Alay Valley in Chon-Alay (Alay district, Osh region, Kyrgyzstan). We (SS, AA, and WT) examined Chegirtke canyon, the Chegirtke mountain range, part of the Kyzyl-Unkur river valley near the village of Kabyk (39.587401°N, 72.387371°E; see Fig 2). We also investigated part of the Altyn-Dara river valley (up to the first border post with Tajikistan in the village of Kary-Shybak) and the river valley Kyok-Suu (10 km north of the village of Zhash-Tilek). In addition to a number of *kurgans* and petroglyphs, we identified habitation structures at three localities (Chegirtke Canyon, Chegirtke Cave, and Kyzyl-Unkur). Recent marmot damage to structures at two of these sites (Chegirtke Canyon and Kyzyl-Unkur) had exposed archaeological ceramics as well as large/medium mammalian bone fragments, which we collected for analysis.

### Excavation

**Fig 3:**
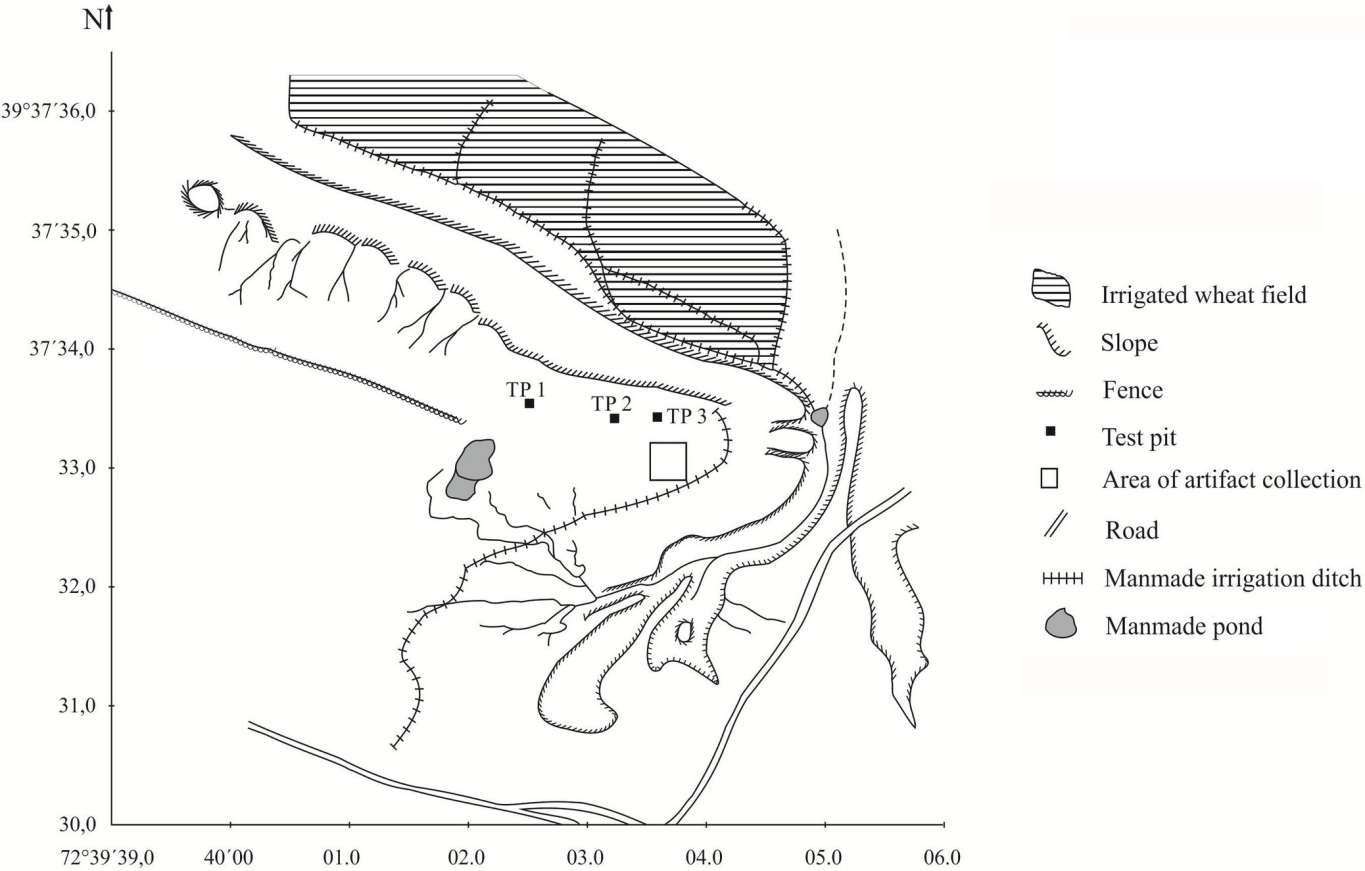
Map of the Alay Site and archaeological test pits/collection area, as they relate to topographical and modern features.

In addition to conducting archaeological survey, we also returned to the location of the Alay Site, near the village of Kashka Suu (Fig 3). An exposed and eroding cultural layer of Paleolithic stone tools was identified, along with small tooth fragments which were collected for analysis. We conducted several test excavations, the results of which were recently published by Shnaider et al. [15,19] These did not yield dateable organic remains.

**Fig 4:**
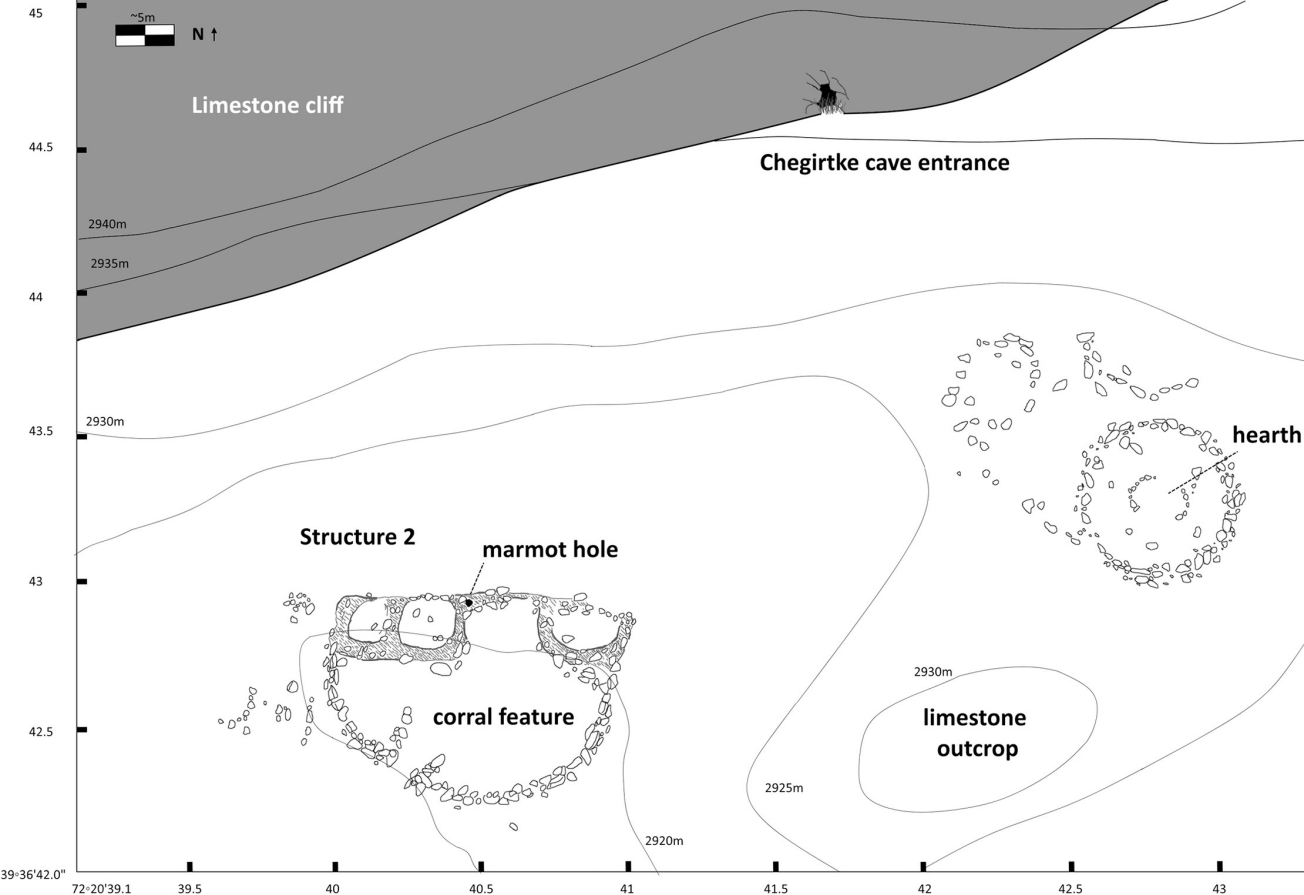
Plan view sketch of Chegirtke Cave entrance and associated habitation structures.

During discussion with local residents in the village of Kabyk, we were alerted to the presence of a cave occasionally used for winter shelter for livestock (Chegirtke Cave). The cave, which is located at the base of a limestone outcrop (Fig 4), currently measures about 10m high and 10m in diameter, although it has been heavily sedimented and a second, smaller chamber can be accessed by crawling. Outside the cave are two habitation structures, one made of stone on an upper terrace (Structure 1), and a lower one made of stone and mudbrick (Structure 2). After visiting and mapping this site and collecting and recording all surface artifacts, we conducted test excavations (0.5x0.5m) within the cave, excavating to a depth of 80 cm.

### Radiocarbon dating

We selected animal bones from each structure that yielded archaeological fauna for radiocarbon dating using Accelerator Mass Spectrometry (AMS). This was performed at the Center for Isotope Research at the University of Groningen, Netherlands. Although deeper areas of Chegirtke Cave Layer 3 did not yield faunal remains, we dated this layer using charcoal. All submitted samples yielded dateable material following chemical pretreatment, which included Acid-Base-Acid (with/without ultrafiltration) for bone samples and Acid-Base-Acid for charcoal.

### Zooarchaeology by Mass Spectrometry

For each specimen, we conducted taphonomic analysis and species identifications using comparative faunal collections held by the Russian Academy of Sciences-Siberia in their field station in Aidarkyen, Kyrgyzstan, and attempted refits for all specimens to control for issues of fragmentation. In addition, we used collagen fingerprinting (or Zooarchaeology by Mass Spectrometry, also known as ZooMS) to taxonomically identify 45 bones from study locations. We demineralized 10 mg of bone using 50mM ammonium bicarbonate (Sigma-Aldrich) pH 8, following the protocol outlined by van Doorn et al [20]. The extracted collagen was digested into component peptides using trypsin (Pierce), and was purified using Pierce C18 tips (Thermo Scientific), eluting in 50% acetonitrile (Sigma-Aldrich) and 0.1% TFA (Sigma-Aldrich). Samples were then spotted on Bruker AnchorChip with Bruker Peptide Calibration Standard in calibration spots directly neighbouring the samples. Mass spectrometric analysis was conducted using a Bruker Autoflex Speed LRF MALDI-TOF in the dedicated ZooMS Laboratory at the Max Planck Institute for the Science of Human History in Jena, Germany. The acquisition used the following parameters: 4000 laser shots at 50–60% intensity (50 shots per spot), mass range 600–3500 Da, reflector mode (additional technical details provided in Text A in S1 File). Identifications were made using published reference spectra from the Eurasian mammals database [21], and are reported according to the level of taxonomic specificity. In some cases, (e.g. sheep vs. muskox and chamois), species that were not necessarily separable on the basis of observed peptide markers were inferred on the basis of known habitat distribution. These are noted with an asterisk. One taxon could not be identified on the basis of published reference data, although we offer inferences based on similarities to available reference spectra. All peptide marker data are provided in Table A in S1 File and additional details regarding MALDI-TOF parameters are provided in Text A in S1 File.

### Genomic analysis

For specimens identified as *Equus* using ZooMS, DNA extractions were carried out at the ancient DNA research facilities of the Laboratoire AMIS CNRS UMR5288, Université de Toulouse III Paul Sabatier. DNA was extracted from 150–300 mg of bone powder following method Y from Gamba et al. [22] with the slight modifications from Gaunitz et al. [23]. Aliquots of DNA extracts were treated with the USER enzyme mix (NEB), following Librado et al. [24] in order to reduce the impact of post-mortem DNA damage in downstream analyses. Triple-indexed DNA libraries were constructed following the methodology from Gaunitz et al. [23] with the exception that the DNA adapters from Rohland et al. [25] (which include 7-bp long internal indexes) were used during the ligation step. We amplified DNA libraries in 25uL reactions using the AccuPrimePfx DNA polymerase following Gaunitz et al. [23]before concentrating them using Agencourt bead purification, quantifying them on a TapeStation 4200 instrument (Agilent technologies) and pooling them together for sequencing on the Illumina Miniseq instrument. Sequencing reads were demultiplexed based on their internal adapter indexes using AdapterRemoval2 [26] before they were parsed through PALEOMIX v1.1.1 [27], in order to identify high-quality reads mapping uniquely against the horse nuclear reference genome EquCab2 [28]and the horse mitochondrial genome (Accession # NC_001640). The resulting read alignment files were then processed with the Zonkey package [29] to identify first-generation equine hybrids and determine the molecular sex of each individual.

### Permits and repository information

This research was conducted under Permit Form 3 issued to A. Abdykanova (Otkrytyi List, Forma 3) by the Archaeological Field Committee of the lnstitute of History and Cultural Heritage at the National Academy of Sciences of the Kyrgyz Republic, who provided permission to conduct archaeological survey and research between April 19 and November 30, 2017. All artifacts (except bone samples submitted for ZooMS analysis) found as a result of survey are stored at the Anthropology Program of American University of Central Asia, Bishkek, Kyrgyzstan, in accordance with Kyrgyz law. Bone samples analyzed in this study are retained in collections at the Max Planck Institute for the Science of Human History in Jena, Germany, and are available for study upon request.

## Results

New radiocarbon results are shown in Table 1. All dates were calibrated using the IntCal13 calibration curve in the program OxCal.

**Table 1:** Radiocarbon dates from 2017 fieldwork in the Alay Valley.

Site	Material	Context	Radiocarbon date	Error	Calibrated date range (95.4%)	Laboratory number
Chegirtke Cave, Layer 3, depth 7	Charcoal	Excavation	9044	18	8290–8250 BCE	GrM-11875
Chegirtke Cave, Layer 3, depth 5	Bone	Excavation	3960	25	2570–2350 BCE	GrM-12292
Chegirtke Cave, Layer 2, depth 3	Bone	Excavation	3718 3686	15 15	2200–2040 BCE 2140–2030 BCE	GrM-11866 GrM-11874
Chegirtke Cave, Layer 2, depth 1	Bone	Excavation	3783	15	2290–2140 BCE	GrM-11872
Kyzyl-Unkur	Bone	Marmot hole	1694	15	260–400 BCE	GrM-11867
Chegirtke Canyon, Structure 2	Bone	Marmot hole	907	15	1040–1170 CE	GrM-11870
Chegirtke Canyon, Structure 1	Bone	Marmot hole	405	15	1440–1490 CE	GrM-11869

### Alay site

At the Alay Site, we discovered dental fragments from a large or medium mammal within a surficial artifact deposit of stone tools and lithic debitage. A tooth specimen was identified as *Ovis* using ZooMS, which may represent the wild *Ovis ammon* or the domesticated *Ovis aries*. Because the specimen was not suitable for radiocarbon dating, it is impossible to determine conclusively whether this specimen was originally associated with the deposit or whether it represents intrusive material. However, typological analysis of stone tools from the site suggests that it dates to between the LGM and the early Holocene, and is analogous to layers 4 and 5 at the site of Obishir-V in the Ferghana Valley [15]. These Obishir-V layers show evidence of specialized sheep/goat consumption, making an association of the present *Ovis* specimens with the archaeological deposits at the Alay site reasonable.

### Chegirtke Cave

Several habitation structures at the site provided a small sample of faunal remains that shed light on the earliest history of animal domesticate use in the Alay Valley. The lowest stratigraphic layer, Layer 3, is a yellowish loess deposit. Charcoal from the bottom of the test pit and an animal bone from the upper portion of this layer provide a direct date to ca. 8250 and ca. 2500 BCE, respectively (Table 1). Due to missing peptide markers, the two bones associated with this layer could not be securely identified, although one of these was morphologically consistent with an ovicaprid scapula (Table A in S1 File). A modified lithic core was found in the upper portions of this layer, which appear to date to the early Bronze Age.

**Fig 5:**
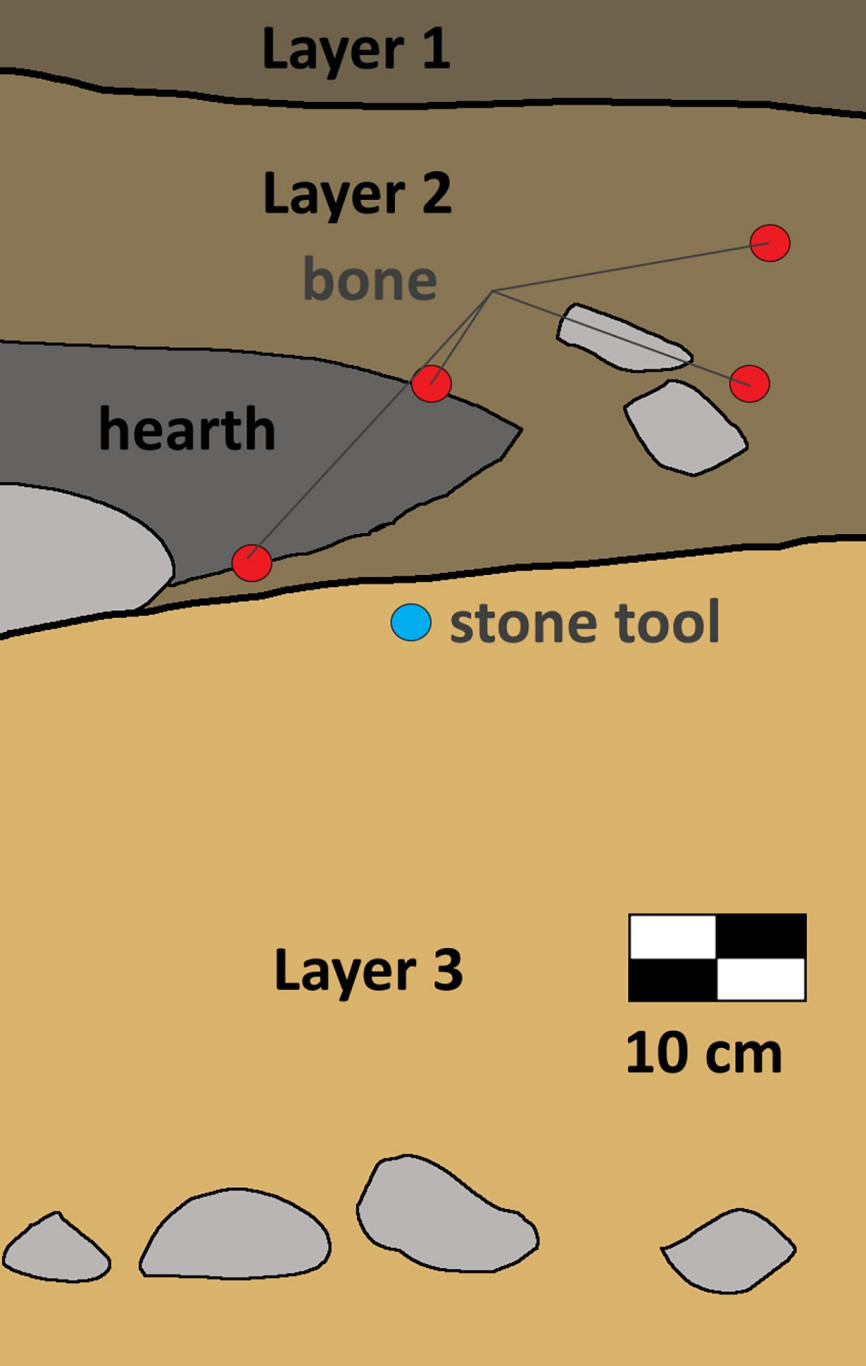
West profile of test Pit 1, Chegirtke Cave. Unit was excavated to a depth of 80 cm, without reaching bedrock. Locations of animal bone within the profile are labeled with red dots, and the stone core tool location is labeled with blue.

The primary cultural component at Chegirtke Cave is a dark organic layer (Layer 2) containing a hearth feature, from which the majority of faunal remains derive (Fig 5). Several ceramic fragments from this layer show “Bronze Age” characteristics, including brown and black coloration and simple incised decorations. Radiocarbon dating of animal bone from various depths in this layer place its occupation at between ca. 2290–2040 BCE, and indicates that the layer represents a single, related assemblage (Table 1). Traditional morphological comparisons revealed only the presence of sheep or goat (n = 5) as well as a lagomorph (n = 1), while most of the fauna assemblage was fractured into long bone pieces of large or medium mammals which could not be further identified to taxon morphologically (Table A in S1 File). The assemblage shows signs of human consumption, including spiral fracturing, burning, and cut marks, indicative of its anthropogenic origins.

**Fig 6:**
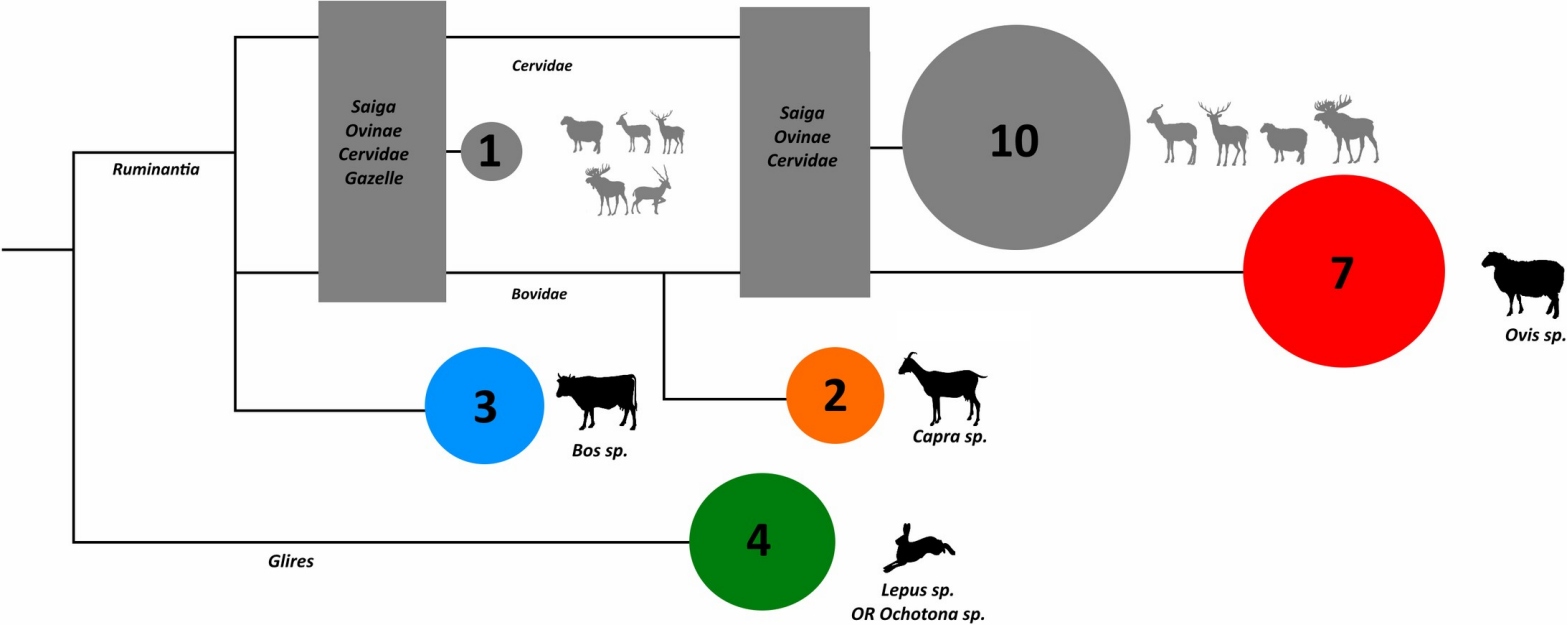
Collagen peptide-based identifications for Layer 2 at Chegirtke, grouped into cladogram by general phylogenetic relationships. Specimens with a grey circle are missing necessary peptide markers for more detailed identification, and silhouettes depict a range of possible taxa that specimens in that category may derive from.

ZooMS enabled a much more refined assessment of these specimens, indicating that the majority indeed represent *Ovis* (n = 7), with smaller numbers of *Bos* (n = 3) and *Capra* (n = 2). Peptide marker C was absent in numerous other analyzed specimens (n = 11), limiting their peptide mass fingerprint assignment to Cervid/Saiga/Ovinae (Fig 6, Table A in S1 File). However, as we only identified *Ovis* sp. through ZooMS and morphological identifications (and one could be morphologically identified as *Ovis/Capra*), it appears likely that these Cervid/Saiga/Ovinae specimens may also represent *Ovis* sp. as well. The co-occurrence of sheep, goat, and cattle remains together suggests that they represent a pastoral assemblage of domestic animals. ZooMS also confirmed the presence of a fourth small or medium-sized mammal species with key similarities to reference spectra for *Lepus*. One of these specimens is spirally fractured, another burned, all are highly fragmented, and most were recovered from within the hearth or cultural level–pointing to an anthropogenic rather than a natural origin. Additional characterization of reference spectra for local fauna will be necessary to narrow down this identification further, although the Tolai hare is relatively common at higher altitudes in this region.

### Kyzyl-Unkur

**Fig 7:**
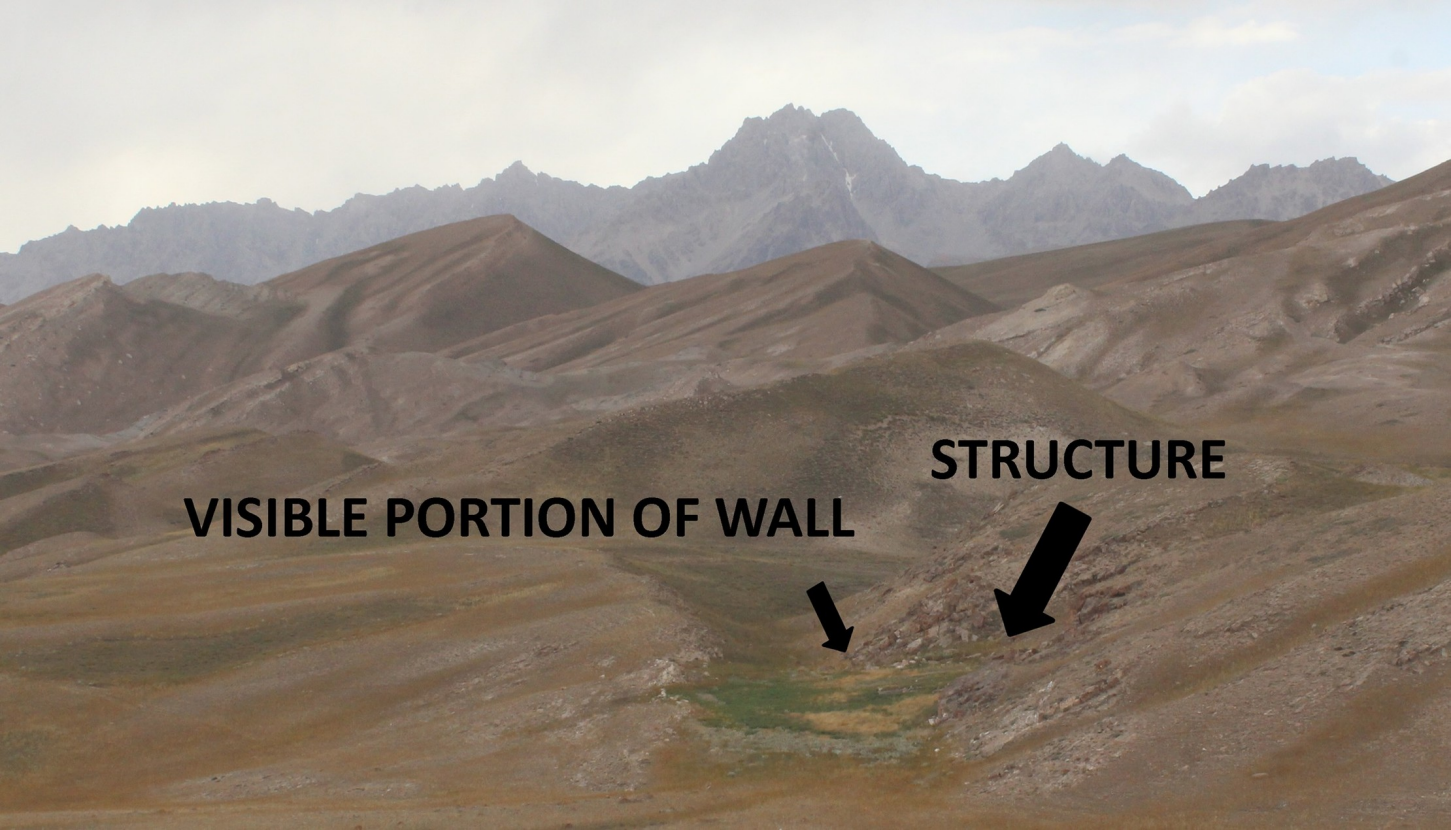
Early Medieval habitation site at Kyzyl-Unkur, showing visible structure wall.

Around six kilometers to the east of Chegirtke Cave, we discovered a series of structures at the site of Kyzyl-Unkur, located in a small notch in the foothills near a large kurgan. The area is well-watered and covered with dense grass vegetation, obscuring some of the architectural layout, but a large stone enclosing wall is visible along the area’s north margin (Fig 7). Marmot activity in the center of the vegetated area has exposed a wall as well as a former hearth, yielding several fragments of reddish ceramic along with two fragments of archaeological animal bone. Neither of the bones could be identified to taxon on the basis of morphological traits. Using ZooMS, the medium fragment was identified as Cervid/Saiga/Ovinae. Similar to our inference made above, the medium fragment could therefore represent a domestic sheep (*Ovis* sp.). ZooMS results identify the large mammal bone as *Equus*. On the basis of available peptide markers and level of analyses, ZooMS is unable to distinguish between the various equids present in Eurasia, which include domestic horse, donkey and wild asses (onager, kulan, kiang). A direct radiocarbon date from the equine bone places the occupation of this structure at ca. 260–400 CE (Table 1).

### Chegirtke canyon

**Fig 8:**
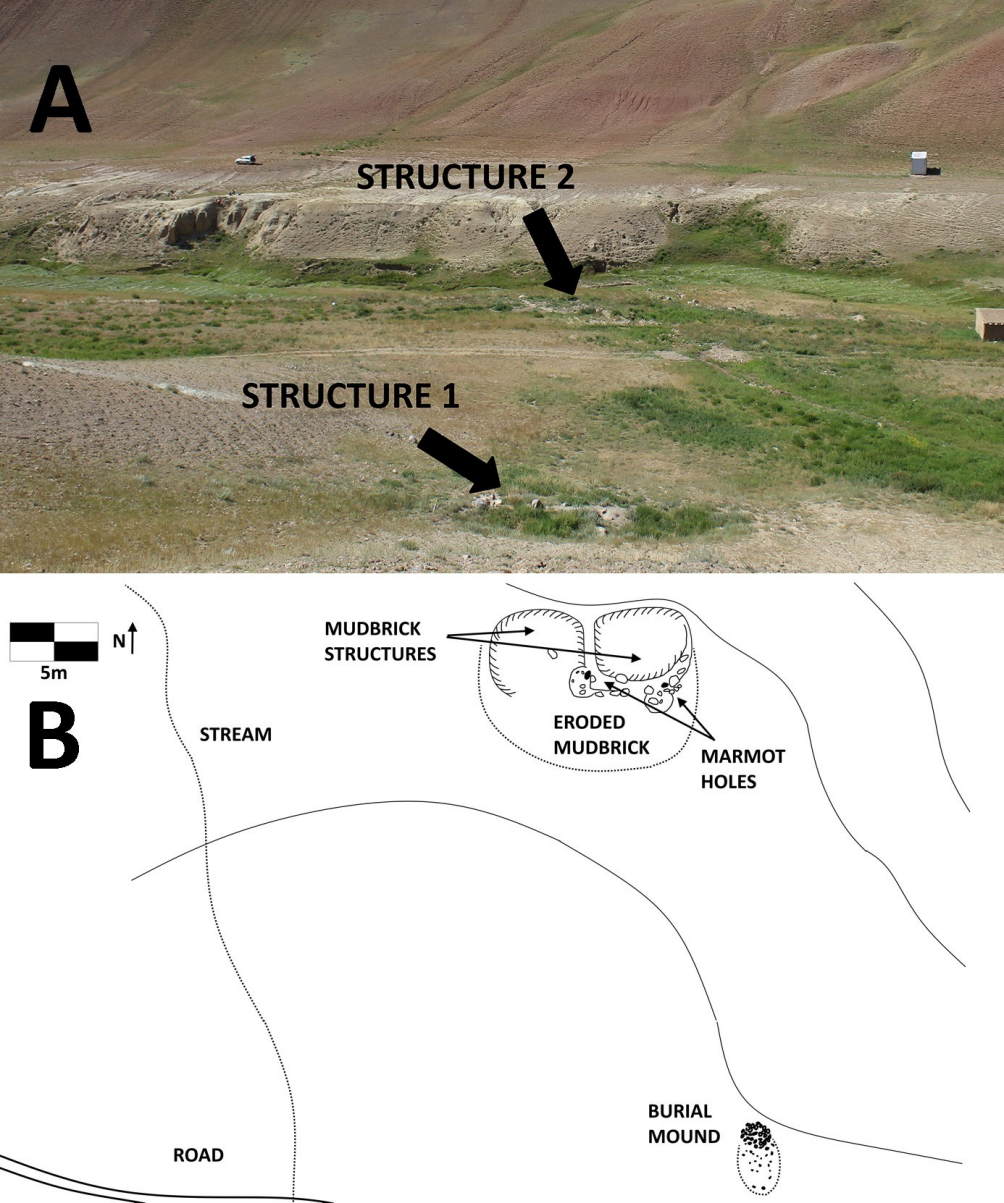
A) View of Chegirtke Canyon Structures 1 and 2, on the northern bank of a small creek. View to south, and B) Plan view sketch of Chegirtke Canyon Structure 2, showing two-room mudbrick structure and location of nearby cultural and topographic features.

In the drainage roughly 2.5 km north/northwest of Chegirtke Cave, also located on the south-facing slopes of the foothills of the Alay range, we discovered a number of small habitation structures concentrated at the well-watered mouth of a mountain runoff channel. The first of these, Structure 1, is located on the upper terrace of the small creek that runs through the canyon, and consists of a two-room mudbrick structure (each room about 5–6 meters in diameter, Fig 8). The second structure, Structure 2, is located adjacent to the creek bottom, and while vegetative cover obscures the architecture, appears to be a single-room mudbrick construction. Recent marmot activity at both structures has upturned archaeological materials, including reddish ceramics, charcoal, a fragment of a small green glass bottle, and a piece of worked wood, along with a variety of animal bone. A radiocarbon date from animal bone recovered from Structure 1 places its construction in the late Medieval period, ca. 1440–1490 CE, while Structure 2 dates several centuries earlier, to ca. 1040–1170 CE.

Like the other sites in the survey area, very little of the recovered animal bone could be identified to taxon based on morphology. Those fragments which retained identifying traits included two specimens of *Ovis/Capra* from Structure 1, one *Ovis/Capra* from Structure 2, and one *Equus* specimen from Structure 2. Application of ZooMS to the small assemblages of animal bones from these two sites greatly improved the identified species diversity, showing that the earlier structure contained one *Ovis* specimen, three Cervid/Saiga/Ovinae, which may represent *Ovis*, two equids, and one specimen of the Bactrian camel (*Camelus bactrianus*), which can be distinguished from dromedary (*Camelus dromedarius*) on the basis of unique peptide markers. The later structure (Str. 1) yielded one specimen definitively identified as *Ovis*, five unidentified Cervid/Saiga/Ovinae, and one equid (Table A in S1 File).

### Equine DNA results

We successfully extracted ancient DNA from all three analysed individuals and recovered 2,713–94,119 endogenous sequences uniquely mapping to the horse reference nuclear and mitochondrial genomes, which represents sufficient data for an identification of the sex and species and/or first-generation hybrid status of each individual through Zonkey [29] Results indicate that all recovered equid specimens are *E*. *caballus* (Table 2), rather than domestic donkey, wild equids, or hybrids between equid taxa, and all were female.

**Table 2:** Genetic identification of species and sex of *Equus* specimens found in Alay archaeological contexts through DNA sequencing.

Specimen	^14^C age	Endogenous DNA content	Mitochondrial fold-coverage	Total number of reads sequenced	Reads uniquely mapping to nuclear reference	Reads uniquely mapping to mitochondrial reference	Species	Sex
Kyzyl-Unkur	263–396 cal. BCE	9.42%	0.45	1,002,938	94,119	127	Horse (*Equus caballus*)	**♀**
Chegirtke Canyon, Structure 2—A75.2	1041–1168 cal. CE	2.52%	0.12	707,407	17,741	32	Horse (*Equus caballus*)	**♀**
Chegirtke Canyon, Structure 2—A74.1		4.49%	0.11	423,540	18,952	31	Horse (*Equus caballus*)	**♀**
Chegirtke Canyon, Structure 1—A72.1	1442–1488 cal. CE	0.34%	0.01	798,411	2,713	2	Horse (*Equus caballus*)	**♀**

## Discussion

This study has several key limitations, including a limited sample size, and the observation that–as is true with morphology-based identification–specimen fragmentation may result in the same individual or the same skeletal element being counted more than once. As a result, our data provide only relative abundance and a comparatively low-resolution proxy for the ancient economies of this critically important but poorly characterized region of the archaeological record. Nonetheless, as with other ZooMS studies of fragmentary bone material, collagen peptide mass fingerprinting of fragmentary faunal remains from Alay adds new insights into the domestic and wild species exploited by ancient peoples in assemblages that may be otherwise largely beyond morphological identification[30–34].

While the absolute number of faunal remains recovered is small, the detail provided by radiocarbon dating, collagen peptide fingerprints, and ancient DNA reveals important information about the history of domesticated animals in a key channel of the ancient Silk Road. During the late Pleistocene or early Holocene, the first human occupants reached the Alay Valley, and appear to have consumed *Ovis* species–perhaps the wild argali sheep, *Ovis ammon*, which is known for its large and impressive local subspecies (the “Marco Polo” sheep).The economy of the early and middle Holocene is not covered by our present dataset, although Layer 3 at Chegirtke shows that people continued to use stone tools and likely consumed either sheep or cervids ca. 2570–2350 BCE. On the basis of this study, it appears that livestock-herding (and likely agropastoral) peoples began to occupy the mountain foothills by at least the early Bronze Age, as evidenced by the cultural layer at Chegirtke Cave, dated to ca. 2290–2040 BCE.

Many influential scholars (e.g. [16,35]) have argued that the spread of pastoralism across Central Asia was likely aided by the use of horses. A key tenet of this argument was based on the assumption that horses have been used for mounted riding since ca. 3500 BCE, as evidenced by tooth damage to horses at the site of Botai in northern Kazakhstan [36] However, recent genomic research indicates that Botai horses were actually the direct ancestors of *E*. *przewalskii*, and were neither the progenitor of modern domestic horses nor those found in domestic contexts within the last ~4,000 years [23]. This finding has prompted the interpretation of Botai as evidence for either a separate, additional horse domestication process whose temporal and geographic locus is currently unknown, or an eventual genomic replacement of the original Botai ancestry through introgression capture as domestic horses expanded and admixed with local populations of wild horses [37]. Resolving this issue will likely require further research. Nonetheless, the earliest direct evidence for the use of the modern domestic horse, *Equus caballus*, in transport now falls to the chariot burials of the Sintashta culture, ca. 2000 BCE (also found in northern Kazakhstan and the Trans-Ural region of southern Russia). Our sample size is too limited to exclude the presence of *Equus* at Chegirtke, and there are viable reasons (seasonality of occupation, exploitation for transport only) that horses might leave a limited archaeofaunal signature even when playing an important economic role [38]. However, radiocarbon dates from Layer 2 at Chegirtke largely predate reliable radiocarbon dates from Sintashta [35]. Considered alongside new findings from Botai, and the apparent absence of horses in other Central Asian pastoral assemblages predating ca. 2000 BCE [39], the argument that horse domestication played a key role in the appearance of early Bronze Age pastoralists in the Alay Valley appears poorly supported by current evidence.

**Fig 9:**
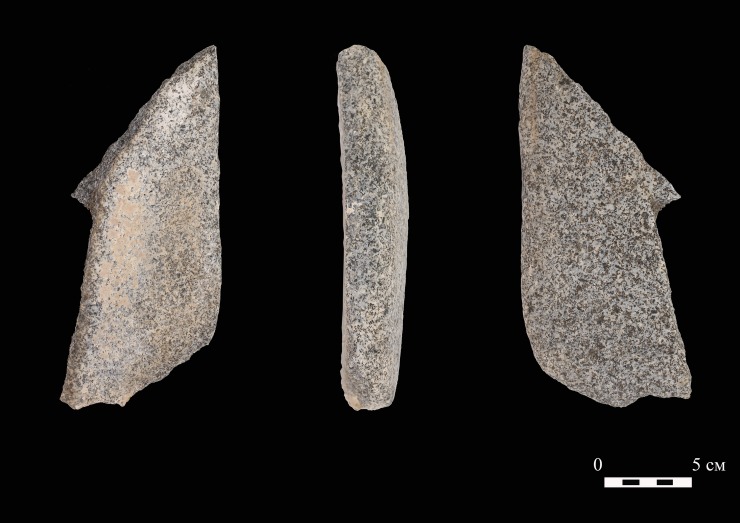
Grinding stone recovered from the surface within Chegirtke Cave, Structure 1.

Across all periods, sheep appear to have been the most important taxon for pastoral subsistence in the Alay Valley. *Ovis* species dominate the small faunal assemblages at Chegirtke Canyon, as well as the remains from nearby Chegirtke Cave. During the early Bronze Age, inhabitants at Chegirtke Cave also herded cattle and goat. Although sample size is small, these form a smaller portion of the assemblage than sheep, a pattern that mirrors other early Bronze Age pastoral assemblages in the mountainous region of Central Asia [39–41], as well as modern herd distributions. In herds documented in Kyrgyzstan in the late 20^th^ century, livestock consisted of more than 86% sheep, 11% cattle, and less than 3% goat [10]. Early Bronze Age occupations at Chegirtke Cave also exploited a wild species of lagomorph (probably the Tolai hare, a wild hare native to the region). This pattern is consistent with other early Bronze Age sites in Central Asia, which show a decreasing proportion of wild fauna over the course of the Bronze Age [42]. The large granitic grinding stone (Fig 9) found within Structure 1 near the entrance to Chegirtke Cave, if temporally associated, suggests that these people practiced a mixed agricultural and pastoral strategy. The stone architecture of both exterior structures would point to a relatively stable, regularized occupation cycle that might be characterized as “semi-sedentary” rather than nomadic. While no direct evidence pertinent to seasonality was recovered from our faunal assemblage, ethnographic observations [10] allow us to suggest that Chegirtke Cave may have been used in the winter, owing to the south-facing slopes and the cave’s protective shelter. The lifeways of these early herders may have played a key role in the formation of networks and pathways that later formed the Silk Road [18].

**Fig 10:**
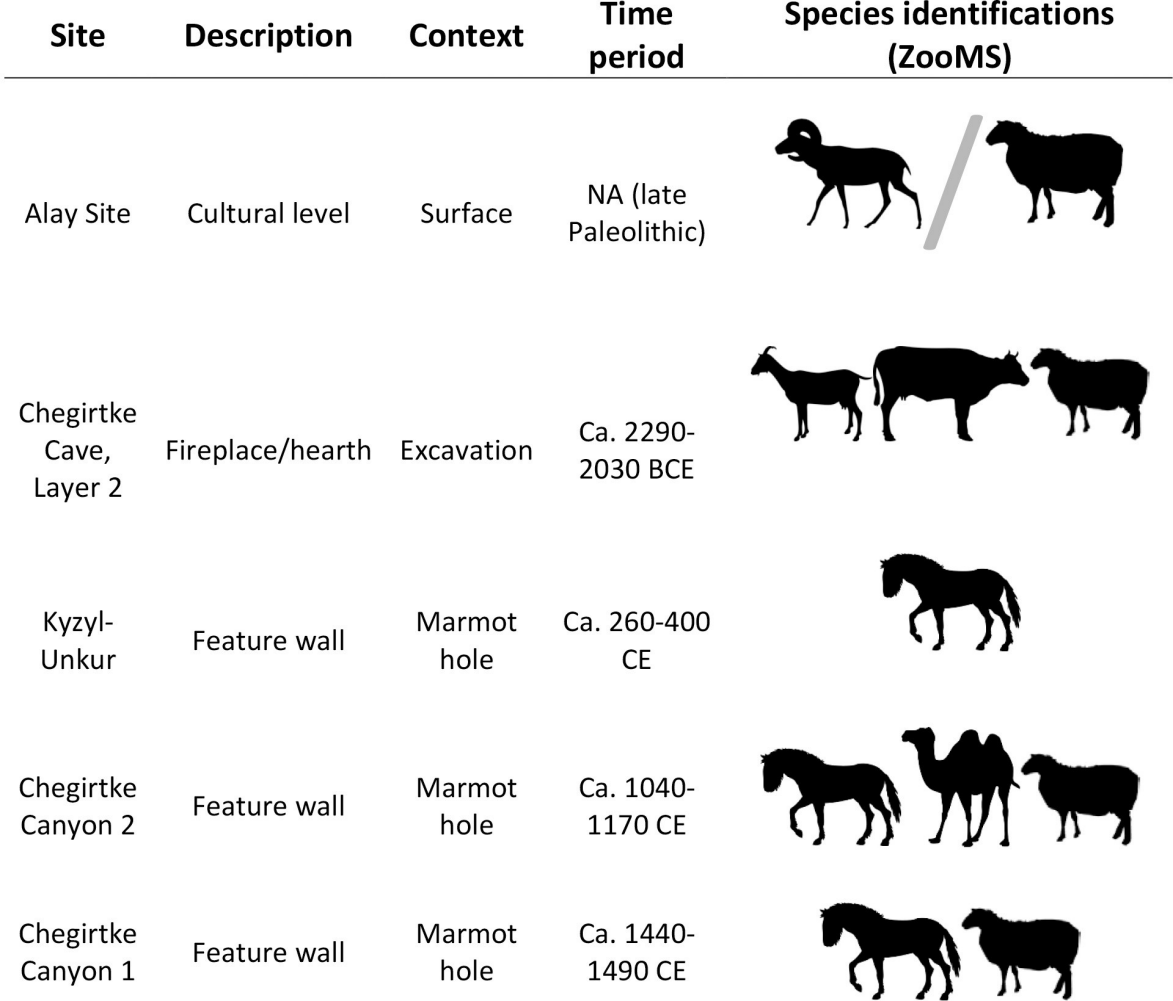
Species richness from ZooMS analysis of faunal remains from habitation structures in the survey area, along with approximate age. Specimens with lower levels of taxonomic specificity have been excluded from this table.

Despite the small sample size available from our survey of later period habitation sites, one apparent pattern is the regular occurrence of equid bones in features from the early through to the late Medieval period, which sits in marked contrast to their absence in analyzed materials from Chegirtke Cave (Fig 10). Discoveries from the site of Aigyrzhal-2 indicate that domestic horses were physically present in the territory of modern Kyrgyzstan by ca. 1600 BCE [17]. With no dated sites in the Alay Valley from this period, it is difficult to make justifiable inferences about how horses might have been used by local residents at this time. However, by the late second or early first millennium BCE, definitive evidence for mounted horseback riding in Central Asia can be found in both the archaeological and historical records [43]. By the end of the first millennium BCE, military incursions to the Ferghana region by the western Han Dynasty formalized the large-scale exchange of horses between Central Asia and Chinese states, a process which continued and accelerated across the Medieval period [3,6,44]. By the time of the occupation of Kyzyl-Unkur and Chegirtke Canyon, the Alay Valley was already an artery of the Silk Road and a major trade corridor, one in which horses may have been key as both a prized commodity as well as a means of traveling and exchanging goods along the mountain corridors of the Pamir, Alay, and Tien Shan. Other equids, particularly donkeys, are also known to have played a crucial role in the transport networks of Silk Road Central Asia due to their stamina and low water requirements [45]. Nonetheless, our DNA results indicate that all identified equids in the present study were female domestic horses (mares). In contrast to stallions (uncastrated male horses) and geldings (castrated male horses) commonly preferred as battle mounts, mares are more docile and easily controlled. In historical records, mares were often preferred by Mongol cavalry on long journeys because of the added sustenance that could be gleaned from the animal’s milk [46]. The consistent presence of female horses in the small assemblage of analyzed Alay sites may relate to their use in long-distance transport across the ancient Silk Road. At Chegirtke Canyon and Kyzyl-Unkur, mares may have also played a role in subsistence, particularly for the production of *kymyz* (fermented mare’s milk) that remains popular in the Alay region today. Bactrian camel also present in the small Chegirtke Canyon assemblage supports the interpretation that transport animals were key to medieval lifeways in the Alay Valley.

## Conclusion

Our results reveal that the Alay Valley, one of the most important geographic corridors of interior Central Asia and a major artery of the historic Silk Road, was occupied by pastoralists or mixed agropastoralists by the early Bronze Age, during the late 3^rd^ millennium BCE. Although recovered faunal remains were highly fragmented and difficult to identify using traditional archaeozoological approaches, the application of Zooarchaeology by Mass Spectrometry (ZooMS) in conjunction with radiocarbon dating and DNA techniques enabled a clearer view into the subsistence choices of ancient peoples in this important region. When considered in chronological context alongside other early Bronze Age archaeological contexts from montane Central Asia, our results suggest that contrary to some hypotheses, horses did not likely play a role in the genesis of pastoral lifeways in southern Kyrgyzstan during the Bronze Age, at least as a dietary resource. Instead, the ancient herding economy seems to have focused largely on sheep, supplemented by cattle and goat, and perhaps agricultural activity–much as it remains today. Following the development of mounted riding and the formation of the Silk Road, both horses and camels may have increased in economic significance as transport animals, and–with horses–as a trade commodity, a process that may explain their increased visibility in some Alay archaeological assemblages. These data reveal a rich and multi-millennial history of domestic animal use in the Alay Valley, and suggest that the development of horse transport and the formation of transcontinental exchange networks had a key influence on the economic choices of high-mountain herders in the continental interior.

## Supporting information

S1 File (Table A) Diagnostic peptide markers and taxonomic identifications by specimen. (Text A) Technical details for MALDI-TOF ZooMS analysis.
(DOCX)
