# Supplementary material for: Early pastoral economies along the Ancient Silk Road: Biomolecular evidence from the Alay Valley, Kyrgyzstan
Source: PLoS One. 2018 Oct 31;13(10):e0205646. doi: 10.1371/journal.pone.0205646 (PMC6209189; doi:10.1371/journal.pone.0205646)
Supplement: S1 File — (Table A) Diagnostic peptide markers and taxonomic identifications by specimen. (Text A) Technical details for MALDI-TOF ZooMS analysis. (DOCX) [file pone.0205646.s001.docx]

**Supplementary information**

S1 Table A. Diagnostic peptide markers and taxonomic identifications by specimen.

| **Specimen ID** | **Site** | **Context** | **Element** | **Taphonomic Notes** | **Taxonomic ID (morphology)** | **Taxonomic ID (ZooMS)** | **P1** | **A** | **A'** | **B** | **C** | **P2** | **D** | **E** | **F** | **F'** | **G** | **G'** |
| --- | --- | --- | --- | --- | --- | --- | --- | --- | --- | --- | --- | --- | --- | --- | --- | --- | --- | --- |
| A48 | Alay Site | Surface | Tooth (enamel plate) | Fragmented (12 pieces) | NA | Ovis sp.* | 1105.6 |  |  | 1427.7 | 1580.8 | 1648.8 | 2131.1 | 2792.3 | 2883.4 |  | 3017.5 | 3033.5 |
| A109 | Chegirtke Cave | Layer 1 | Radius – R | Spiral fracture | Ovis/Capra | Cervid/Saiga/Ovinae | 1105.6 |  | 1196.6 | 1427.7 |  | 1648.8 | 2131.1 | 2792.3 | 2883.4 | 2899.4 | 3017.5 | 3033.5 |
| A110 | Chegirtke Cave | Layer 2.1 | Cranial fragment | Cut marks | Large mammal | *Bos sp.* | 1105.6 | 1192.7 | 1208.7 | 1427.7 |  | 1648.8 | 2131.1 | 2792.3 | 2853.4 |  | 3017.5 | 3033.5 |
| A111.3 | Chegirtke Cave | Layer 2.1 | Long bone fragment | Spiral fracture | Large mammal | *Bos sp.* | 1105.6 |  | 1208.7 | 1427.7 |  | 1648.8 | 2131.1 |  | 2853.4 |  | 3017.5 | 3033.5 |
| A111.4 | Chegirtke Cave | Layer 2.1 | Long bone fragment | Spiral fracture | Large mammal | *Bos sp.* | 1105.6 |  | 1208.7 | 1427.7 |  | 1648.8 | 2131.1 | 2792.3 | 2853.4 |  | 3017.5 | 3033.5 |
| A111.1 | Chegirtke Cave | Layer 2.1 | Long bone fragment | Spiral fracture | Medium mammal | Cervid/Saiga/Gazelle/Ovinae | 1105.6 |  |  | 1427.7 |  |  | 2131.1 | 2792.3 | 2883.4 |  | 3017.5 | 3033.5 |
| A111.2 | Chegirtke Cave | Layer 2.1 | Long bone fragment | Carbonized, >50% | Large/medium mammal | Cervid/Saiga/Ovinae | 1105.6 |  |  | 1427.7 |  | 1648.8 | 2131.1 | 2792.3 | 2883.4 | 2899.4 | 3017.5 | 3033.5 |
| A111.5 | Chegirtke Cave | Layer 2.1 | Scapula fragment | --- | Medium mammal | Cervid/Saiga/Ovinae | 1105.6 |  |  | 1427.7 |  | 1648.8 | 2131.1 | 2792.3 | 2883.4 |  | 3017.5 | 3033.5 |
| A111.7 | Chegirtke Cave | Layer 2.1 | --- | --- | --- | Cervid/Saiga/Ovinae | 1105.6 |  |  | 1427.7 |  | 1648.8 | 2131.1 | 2792.3 | 2883.4 |  | 3017.5 | 3033.5 |
| A111.9 | Chegirtke Cave | Layer 2.1 | --- | --- | --- | Cervid/Saiga/Ovinae | 1105.6 | 1180.6 |  | 1427.7 |  | 1648.8 | 2131.1 | 2792.3 | 2883.4 | 2899.4 | 3017.5 | 3033.5 |
| A111.10 | Chegirtke Cave | Layer 2.1 | --- | --- | Small mammal | Glires (Lagomorpha/Rodentia) | 1105.6 | 1221.6 | 1235.6 | 1453.3 |  |  | 2129.1 | 2808.3 | 2883.4 | 2899.4 | 2957.4 |  |
| A111.6 | Chegirtke Cave | Layer 2.1 | Proximal epiphysis, humerus (unfused) | --- | Lagomorpha | Glires (Lagomorpha/Rodentia) | 1105.6 |  |  | 1453.3 |  |  | 2129.1 | 2808.3 | 2883.4 |  | 2957.4 |  |
| A111.8 | Chegirtke Cave | Layer 2.1 | Long bone fragment | Spiral fracture | Small mammal | Glires (Lagomorpha/Rodentia) | 1105.6 | 1221.6 |  | 1453.3 |  |  | 2129.1 | 2808.3 | 2883.4 | 2899.4 | 2957.4 |  |
| A117.1 | Chegirtke Cave | Layer 2.2 | Cranial fragment | --- | Ovis/Capra | *Capra sp.* | 1105.6 | 1180.6 | 1196.6 | 1427.7 |  | 1648.8 | 2131.1 | 2792.3 | 2883.4 |  | 3077.5 | 3093.5 |
| A117.2 | Chegirtke Cave | Layer 2.2 | Long bone fragment | Spiral fracture | Large/medium mammal | *Capra sp.* | 1105.6 |  | 1196.6 | 1427.7 |  | 1648.8 | 2131.1 | 2792.3 | 2883.4 | 2899.4 | 3077.5 | 3093.5 |
| A112.1 | Chegirtke Cave | Layer 2.2 | Long bone fragment | Carbonized, <50%  Spiral fracture | Medium mammal | Cervid/Saiga/Ovinae | 1105.6 |  | 1196.6 | 1427.7 |  | 1648.8 | 2131.1 | 2792.3 | 2883.4 | 2899.4 | 3017.5 | 3033.5 |
| A113.3 | Chegirtke Cave | Layer 2.3 | Metapodial fragment | --- | Medium mammal | Cervid/Saiga/Ovinae | 1105.6 | 1180.6 | 1196.6 | 1427.7 |  | 1648.8 | 2131.1 | 2792.3 | 2883.4 | 2899.4 | 3017.5 | 3033.5 |
| A114.2 | Chegirtke Cave | Layer 2.3 | Flat bone fragment | --- | Medium mammal | Cervid/Saiga/Ovinae | 1105.6 | 1180.6 |  | 1427.7 |  | 1648.8 | 2131.1 | 2792.3 | 2883.4 | 2899.4 | 3017.5 | 3033.5 |
| A114.3 | Chegirtke Cave | Layer 2.3 | Long bone fragment | Carbonized, >50% | Medium/small mammal | Glires (Lagomorpha/Rodentia) | 1105.6 | 1221.6 |  | 1453.3 |  |  | 2129.1 | 2808.3 | 2883.4 | 2899.4 | 2957.4 |  |
| A113.1 | Chegirtke Cave | Layer 2.3 | Proximal radius fragment (R) | Spiral fracture | Ovis/Capra | Ovis sp.* | 1105.6 | 1180.6 | 1196.6 | 1427.7 | 1580.8 | 1648.8 | 2131.1 | 2792.3 | 2883.4 | 2899.4 | 3017.5 | 3033.5 |
| A113.2 | Chegirtke Cave | Layer 2.3 | Sternum | Carnivore tooth puncture | Ovis/Capra | Ovis sp.* | 1105.6 |  |  | 1427.7 | 1580.8 | 1648.8 | 2131.1 | 2792.3 | 2883.4 | 2899.4 | 3017.5 | 3033.5 |
| A113.4 | Chegirtke Cave | Layer 2.3 | Cranial fragment | --- | Medium mammal | Ovis sp.* | 1105.6 |  |  | 1427.7 | 1580.8 | 1648.8 | 2131.1 | 2792.3 | 2883.4 | 2899.4 | 3017.5 | 3033.5 |
| A114.1 | Chegirtke Cave | Layer 2.3 | Cranial fragment | --- | Medium mammal | Ovis sp.* | 1105.6 |  | 1196.6 | 1427.7 | 1580.8 | 1648.8 | 2131.1 | 2792.3 | 2883.4 | 2899.4 | 3017.5 | 3033.5 |
| A115.1 | Chegirtke Cave | Layer 3.5 | Scapula fragment (L) | --- | Ovis/Capra | Cervid/Saiga/Ovinae | 1105.6 |  |  | 1427.7 |  | 1648.8 | 2131.1 | 2792.3 | 2883.4 |  | 3017.5 | 3033.5 |
| A115.2 | Chegirtke Cave | Layer 3.5 | Cranial fragment | --- | Medium mammal | Cervid/Saiga/Ovinae | 1105.6 |  |  | 1427.7 |  | 1648.8 | 2131.1 | 2792.3 | 2883.4 |  | 3017.5 | 3033.5 |
| A108.1 | Chegirtke Cave | Wall cleaning, TP1 | --- | --- | Medium mammal | Ovis sp.* | 1105.6 | 1180.6 | 1196.6 | 1427.7 | 1580.8 | 1648.8 | 2131.1 |  | 2883.4 |  | 3017.5 | 3033.5 |
| A108.2 | Chegirtke Cave | Wall cleaning, TP2 | --- | --- | Medium mammal | Ovis sp.* | 1105.6 | 1180.6 |  | 1427.7 | 1580.8 | 1648.8 | 2131.1 | 2792.3 | 2883.4 | 2899.4 | 3017.5 | 3033.5 |
| A108.3 | Chegirtke Cave | Wall cleaning, TP3 | Vertebra | Trampling | Medium/small mammal | Glires (Lagomorpha/Rodentia) | 1105.6 |  | 1221.6 | 1453.3 |  |  | 2129.1 | 2808.3 | 2883.4 | 2899.4 | 2957.4 |  |
| A112.2 | Chegirtke Cave | Layer 2.2 | Cranial fragment | Carbonized (100%) | Medium mammal | No sample run |  |  |  |  |  |  |  |  |  |  |  |  |
| A81 | Kyzyl Unkur | Habitation | Long bone fragment | Spiral fracture | Large mammal | *Equus sp.* | 1105.6 | 1182.6 | 1198.6 | 1427.7 |  |  | 2145.1 | 2820.4 | 2883.4 | 2899.4 | 2983.5 | 2999.5 |
| A82 | Kyzyl Unkur | Habitation | --- | Trampling | Medium mammal | Cervid/Saiga/Ovinae | 1105.6 |  | 1196.6 | 1427.7 |  | 1648.8 | 2131.1 | 2792.3 | 2883.4 | 2899.4 | 3017.5 | 3033.5 |
| A74.2 | Chegirtke 1 | Structure 2 | Long bone fragment | Spiral fracture, possible cut marks | Large mammal | *Camelus bactrianus* | 1105.6 | 1221.6 |  | 1443.7 |  | 1634.8 | 2131.1 | 2820.4 | 2883.4 | 2899.4 | 2975.5 | 2991.5 |
| A74.3 | Chegirtke 1 | Structure 2 | Cranial fragment | Trampling | Large/medium mammal | Cervid/Saiga/Ovinae | 1105.6 |  | 1196.6 | 1427.7 |  | 1648.8 | 2131.1 | 2792.3 | 2883.4 | 2899.4 | 3017.5 | 3033.5 |
| A74.4 | Chegirtke 1 | Structure 2 | Flat bone fragment | Trampling | Medium mammal | Cervid/Saiga/Ovinae | 1105.6 |  |  | 1427.7 |  | 1648.8 | 2131.1 | 2792.3 | 2883.4 | 2899.4 | 3017.5 | 3033.5 |
| A74.5 | Chegirtke 1 | Structure 2 | Long bone fragment | Root etching <50% | Medium mammal | Cervid/Saiga/Ovinae | 1105.6 | 1180.6 |  | 1427.7 |  | 1648.8 | 2131.1 | 2792.3 | 2883.4 | 2899.4 | 3017.5 | 3033.5 |
| A74.1 | Chegirtke 1 | Structure 2 | Mandible fragment (R) | Spiral fracture | *Equus sp* | *Equus sp.* | 1105.6 | 1182.6 |  | 1427.7 |  |  | 2145.1 | 2820.4 | 2883.4 |  | 2983.5 | 2999.5 |
| A75.2 | Chegirtke 1 | Structure 2 | Tooth enamel fragment | Trampling | Large/medium mammal | *Equus sp.* | 1105.6 | 1182.6 | 1198.6 | 1427.7 | 1550.8 | 1649.8 | 2145.1 | 2820.4 | 2883.4 | 2899.4 | 2983.5 | 2999.5 |
| A75.1 | Chegirtke 1 | Structure 2 | Lower 3rd premolar | Trampling | Ovis/Capra | Ovis sp.* | 1105.6 | 1180.6 | 1196.6 | 1427.7 | 1580.8 | 1648.8 | 2131.1 | 2792.3 | 2883.4 | 2899.4 | 3017.5 | 3033.5 |
| A70.1 | Chegirtke 1 | Structure 1 | Humerus (R) | Spiral fracture | Ovis/Capra | Cervid/Saiga/Ovinae | 1105.6 |  |  | 1427.7 |  | 1648.8 | 2131.1 | 2792.3 | 2883.4 | 2899.4 | 3017.5 | 3033.5 |
| A70.2 | Chegirtke 1 | Structure 1 | Long bone fragment | Spiral fracture | Large/medium mammal | Cervid/Saiga/Ovinae | 1105.6 |  | 1196.6 | 1427.7 |  | 1648.8 | 2131.1 | 2792.3 | 2883.4 | 2899.4 | 3017.5 | 3033.5 |
| A71 | Chegirtke 1 | Structure 1 | Long bone fragment | Spiral fracture, discoidal (impact) fracture | Medium mammal | Cervid/Saiga/Ovinae | 1105.6 |  |  | 1427.7 |  | 1648.8 | 2131.1 | 2792.3 | 2883.4 | 2899.4 | 3017.5 | 3033.5 |
| A72.2 | Chegirtke 1 | Structure 1 | --- | --- | Large/medium mammal | Cervid/Saiga/Ovinae | 1105.6 |  |  | 1427.7 |  | 1648.8 | 2131.1 | 2792.3 | 2883.4 | 2899.4 | 3017.5 | 3033.5 |
| A78 | Chegirtke 1 | Structure 1 | Rib | --- | Medium mammal | Cervid/Saiga/Ovinae | 1105.6 |  |  | 1427.7 |  | 1648.8 | 2131.1 | 2792.3 | 2883.4 |  | 3017.5 | 3033.5 |
| A72.1 | Chegirtke 1 | Structure 1 | Flat bone fragment | --- | Large/medium mammal | *Equus sp.* | 1105.6 |  |  | 1427.7 |  |  | 2145.1 | 2820.4 | 2883.4 | 2899.4 | 2983.5 | 2999.5 |
| A68 | Chegirtke 1 | Structure 1 | Ulna (L) | Refit (4 fragments) | Ovis/Capra | *Ovis sp.** | 1105.6 |  |  | 1427.7 | 1580.8 | 1648.8 | 2131.1 | 2792.3 | 2883.4 |  | 3017.5 | 3033.5 |

S1 Text A. Technical details for MALDI-TOF ZooMS analysis.

Reflector 3.0x 1917V

Smartbeam Parameter Set 4_large, frequency 2000Hz

Sample Rate 5.00GS/s

Realtime smoothing Off

Baseline Offset Adjustment 0.0%

Analog offset 1.2mV

Matrix suppression: Deflection up to 450 Da

Processing Method SC_Peptide_Cent

Ion Source 1: 18.88kV

Ion Source 2: 16.53 kV

Lens: 8.52 kV

Reflector: 21 kV

Reflector 2: 9.6 kV

Laser: Global Attenuator Offset 25%

Digitizer sensitivity: 100mV

Trigger level: 800mV

Detector Gain Voltages: Linear base 2500V, Reflector Base 1700V, Reflector Boost 50V
